# Current trends in extracellular vesicle research on neuroscience from ADPD2021 meeting

**DOI:** 10.20517/evcna.2021.08

**Published:** 2021-03-30

**Authors:** Tsuneya Ikezu

**Affiliations:** Laboratory of Molecular NeuroTherapeutics, Department of Neuroscience, Mayo Clinic Florida, Jacksonville, FL 32224, USA.

This year’s ADPD2021 meeting (https://adpd.kenes.com) for Alzheimer’s and Parkinson’s disease (AD/PD) research is being held virtually in Barcelona, Spain. There are hundreds of papers for the cutting-edge research and clinical trials on AD/PD. Among them, exciting extracellular vesicle (EV)-related papers were presented by the following scientists from the US, France, Israel, and Spain. Blood neuron and astrocyte-specific EVs are useful liquid biopsy tools for the diagnosis of brain atrophy and cognitive dysfunction in AD/PD (Kapoginiasis), which was validated using multiple animal models of AD (Delgado-Peraza). PD cerebrospinal fluid (CSF)-derived EVs contains phosphorylated alpha-synuclein (a-syn) and can cause PD-like pathology and behavioral deficit after their intranasal administration (Herman). Frontotemporal dementia (FTD) CSF-derived EVs contain unique miRNAs, which can modulate neuronal autophagy activities (Cervera-Carles). Interstitial fluid (ISF)-derived EVs from tau mouse and human AD brains show potent tau seeding activities (Leroux). ISF-EVs show tau seeding activity when isolated from AD cases but not from controls, progressive supranuclear palsy (PSP) cases, or Pick disease (PiD) cases (Colin). Brain-derived EVs from prodromal AD and AD cases have potent tau propagation activity in aged mouse brain and cause neurophysiological dysfunction (Ruan). EV production is increased by aging in female but not male mouse brain, whereas mitochondria-derived EVs (coined as “mitovesicles”) are commonly increased by aging (Kim). Finally, mitovesicles contain mitochondria-specific marker Tomm20 and are released by inhibition of mitochondrial activity. Both animal models and human cases of Down syndrome show enhanced production of mitovesicles in brain, suggesting the mitochondrial dysfunction (D’Acunzo).

Dimitrios Kapoginiasis at National Institute of Aging, NIH presented a comprehensive body of his work on insulin signaling biomarkers in blood exosomes of AD and PD patients. This is based on previous reports that pSer312 and pSer616 insulin receptor substrate-1 (IRS-1) is upregulated in the neurons in Alzheimer’s disease and mild cognitive impairment (MCI) cases. Accordingly, blood L1CAM+ neuron-derived EVs (NEVs) contain increased amount of pIRS-1 in addition to Abeta42, pT181 tau, pT231 tau, and total tau. The team examined NEVs from 350 patients in The Baltimore Longitudinal Study of Aging cohort from 1958 to 2019. In this study, pY-IRS-1 was also significantly elevated in AD NEVs compared to control NEVs. Both pT181tau and pS312-IRS-1 in NEVs are negatively correlated with multiple cognitive functional readouts. In addition, pY-IRS-1 is positively correlated with temporal grey matter volume. This is in line with their recent study demonstrating the positive correlation of pIGF1R, pIR, pY-IRS1, pGSK3B, and p-p70S6K in NEVs with the temporal grey matter volume and negative correlation with the white matter hyperintensities volume. Finally, pY-IRS-1 in NEVs is decreased in individuals with PD and cognitive decline and is negatively correlated with motor symptom severity in PD, suggesting the broader applicability of NEVs as prognostic biomarkers for brain atrophy and cognitive function in AD and PD cases.

Shay Herman from Professor Offen’s lab at Tel-Aviv University, Israel, presented her research on intranasal administration of CSF-derived exosomes from PD patients, which induces PD-like symptoms and pathology. This study was based on previous studies demonstrating the development of alpha-synuclein (a-syn) aggregation after treatment of neurons with CSF-derived exosomes from PD patients in vitro. Intravenous or intrastriatal injections of exosomes derived from PD patient serum or brain lysates of dementia with Lewy bodies were also shown to develop protein aggregation in the substantia nigra (SN) and motor deficits *in vivo*. The EVs were isolated from the patient’s CSF by ultracentrifugation. Exosomal markers (CD81, CD9, and Tsg101) and a-syn (total and pSer129) were detected in the isolated CSF-EVs. Incubation of SH-SY5Y cells with CSF-EVs show significant accumulation of pSer129 a-syn and neurotoxicity in vitro. For the* in vivo* study, C57BL/6 mice received intranasal administration of PD CSF-EVs at three months of age and were tested for behavioral changes at 3-8 months after the treatment. PD CSF-EV-treated mice showed significant motor deficits, elevated anxiety, hyposmia, earlier greying of hair (possible melanin deficiency), and pSer129 a-syn aggregation in the SN. This would be the first demonstration of modeling a-syn aggregation in the SN and motor deficits after intranasal inoculation of PD CSF-EVs in mice.

Laura Cervera-Carles at Ana Maria Cuervo laboratory, Albert Einstein College of Medicine, presented her recent work on characterization of miRNAs in EVs isolated from CSF of patients with FTD, who show protein accumulation of TDP-43 or tau in the frontotemporal region of the brain. They focused on miRNAs in CSF-derived EVs as several FTD-related proteins could interfere with their biogenesis and several miRNA species have been linked to tau phosphorylation and splicing. In their study, EVs were isolated from CSF by precipitation method and verified by cryo-electron microscopy and bead-based flow cytometry for exosomal markers. In total, 752 miRNAs were analyzed by qPCR human panels and 103 miRNAs were detected in the isolated EVs. They initially detected eight potential candidate miRNAs on FTD patient CSF-derived EVs and validated the findings using CSF samples collected from 142 subjects (TDP, TDP/tau, and tau groups). miR-146 and miR-361 were upregulated in the tau FTD group, whereas miR-15 and miR-708 were downregulated or undetectable in the tau FTD group. The research team also investigated the effect of miRNAs on protein turnover using murine neuronal N2a cells expressing human WT tau or P301L mutant tau. Using two fluorescent reporter systems to monitor chaperone-mediated autophagy and macroautophagy dynamics, they concluded that protein degradation appears altered when certain miRNAs and tau forms are co-present in the cell lines, and the effect is dependent on autophagy: co-presence of miRNAs alterations and mutant tau appear to inhibit macroautophagy, whereas P301L mutant tau seems to prevent the activation of chaperone-mediated autophagy by miRNAs. Further studies are necessary to elucidate the molecules involved in the autophagy process regulated by these specific miRNAs.

Elodie Leroux from Luc Buee laboratory at INSERM/Universite de Lillle presented the EV and prion-like spreading in AD. EVs were isolated by size-exclusion column (SEC) from interstitial fluid (ISF), CSF, and plasma. ISF was isolated from WT, Tau30, and APP/PS1 mice. Then, EVs’ isolation was validated for their size by NTA and their morphology by electron microscopy and Western blotting of EV markers. The seeding activity of EVs was determined by the FRET-based tau seeding assay system. The FRET signal is significantly increased in ISF-derived EVs (ISV-EVs) from Thy-tau30 mice at three and six months of age. Immunoprecipitation of tau+ ISF-EVs by HT7 anti-tau antibody reduced the tau seeding activity. ISF-EVs were next isolated from prefrontal lobe of healthy controls and AD cases by SEC. The AD ISF-EVs only also had tau seeding activity, as determined by the FRET-based reporter system. However, AD CSF- or plasma-derived EVs showed no tau seeding activity, presumably due to the sensitivity issue. Overall, this is a significant work to demonstrate the tau seeding activity of ISF-EVs from tau mice, which is elevated by aging. These findings were also reproduced using ISF-EVs from AD cases.

Morvane Colin from Lille Neuroscience & Cognition, France, presented the role of brain EV in tauopathies. They examined the EVs isolated from human ISF (ISF-EVs) using different brain tissues of healthy controls and AD, progressive supranuclear palsy, and Pick disease cases by SEC. There was no difference in the concentration of EVs or tau levels in ISF-EVs among groups or brain regions. Significant tau seeding activity was observed in ISF-EVs isolated from prefrontal cortex, occipital cortex, or cerebellum of AD cases but not from other groups. There was no tau pathology in the cerebellum of the tested AD cases. Finally, ISF-EVs isolated from AD or control cases were injected into Thy-Tau30 mice or non-Tg littermates. MC1 tau accumulation was observed in the CA1 region of AD ISF-EV-injected Thy-Tau30 mice but not by control ISF-EV injection or in non-Tg littermates. These data demonstrate that tau is physiologically secreted in ISF-EVs in all tested groups, but only AD ISF-EVs show tau seeding activity both in vitro and *in vivo* when using Thy-Tau30 mice. Further studies are necessary to understand the difference in tau seeding activity of ISF-EVs among AD, PSP, and PiD cases.

Zhi Ruan from Ikezu lab presented the spread of tau pathology after intrahippocampal injection of EVs isolated from the frontal cortical grey matter of HC, prodromal AD (pAD), and AD cases. Both pAD and AD brain-derived EVs were enriched in tau oligomer species and globular tau, which were mostly present on the intraluminal side of EVs. Proteomic analysis and validation with Western blotting demonstrate the enrichment of glia-specific markers and reduction in neuron-specific markers in AD brain-derived EVs. Both pAD and AD EVs showed enhanced neuronal uptake, but AD EVs also exhibited higher tau seeding activity, as determined by the FRET biosensor system in vitro. Significant tau propagation was observed after hippocampal injection of pAD or AD EVs, which was mainly in GAD67+ interneurons and GluR2/3+ mossy cells in the hilus region. This was accompanied by reduced neuronal activities, as determined by the reduction in GAD67+ synaptic puncta around CA1 pyramidal neurons and c-fos expression, and the enhancement of excitatory/inhibitory activity balance, as determined by the whole-cell patch clamp recording of CA1 pyramidal neurons. This is the first comprehensive demonstration that AD brain-derived EVs propagate tau pathology and induce CA1 neurophysiological dysfunction. This work was recently published in Brain^[1]^. 

Francheska Delgado-Peraza from Kapogiannis lab at NIH presented their work demonstrating that plasma neuronal EVs from multiple AD mouse models contain high levels of total tau, pT181-tau, and Aβ42, which are significantly correlated with their levels in the brain tissues. 2xTg-AD (APP/PS1), 5xFAD, and 3xTg-AD mouse models were used for brain tissue analysis and isolation of EVs from blood using immunoprecipitation for neuron- and astrocyte-specific EVs (NEVs and AEVs) targeting L1CAM and GLAST, respectively. The isolated NEVs were enriched with L1CAM, GRIA2, and conventional exosomal markers. They also showed that circulating astrocytic EVs contain high levels of C1q complement, reflecting brain levels. These findings are direct validation of circulating EVs as indicators of the accumulation of total tau, pT181 tau, Aβ42, and C1q using AD mouse brain and blood samples and demonstrated that NEVs and AEVs have great potential for liquid biopsy modules for AD biomarkers.

Yohan Kim from Efrat Levy lab (Nathan S. Kline Institute for Psychiatric Research, New York, US) presented how sex and aging alter EV secretion in the brain. Brain-derived EVs were isolated from male and female C57BL/6 mice at 3-24 months of age by their established protocol using sucrose gradient ultracentrifugation. The purity was assessed by EM and Western blotting of EV and non-EV markers. EM-based morphological analysis shows no obvious changes in the shape of isolated EVs by aging. Interestingly, the level of annexin-A2 was significantly increased in female but not male brain-derived EVs by aging. This was accompanied by significant increases of Alix, Tsg101, and HSC70 levels only in female brain-derived EV by aging. Another exosome marker (CD63) and a mitovesicle marker (VDAC) were significantly increased by aging in both sexes, suggesting the elevation of specific EVs only in the female brain and mitovesicles as commonly elevated EVs by aging. These data demonstrate the sexual dimorphism of age-dependent increase in EV production in female mouse brain and that mitovesicles can serve as a common indicator of age-related EVs. 

Pasquale D’Acunzo from Dr. Efrat Levy’s lab (Nathan S. Kline Institute for Psychiatric Research, New York, US) presented a study in which the authors described a newly identified population of mitochondria-derived EVs that they coined “mitovesicles”. These EVs are fractionated at the bottom of an OptiPrep density gradient from mouse and human brain tissues and contain an electron-dense matrix and a double membrane when visualized by cryoEM. Proteomic profiling of the mitovesicle fraction showed enrichment of proteins involved in catabolic pathways, including energy production and neurotransmitter/amino acid metabolism. Western blotting analysis of isolated mitovesicles showed enrichment of specific mitochondrial markers but a lack of other common mitochondrial proteins such as Tomm20. EVs isolated from human primary fibroblasts treated with the mitochondrial inhibitor antimycin-A showed increased levels of mitochondria markers, suggesting that mitochondrial dysfunction enhances mitovesicle release. Finally, higher levels of mitovesicles were detected in EVs isolated from a mouse model of Down syndrome, the Ts2 model, and human Down syndrome brain tissues when compared to the respective controls. The work from D’Acunzo *et al*.^[2]^, recently published in *Science Advances*, indicates that mitochondrial dysfunction can be monitored by the amount of mitovesicles *in vivo* and that the status of intracellular mitochondria can be inferred by the analysis of mitovesicle cargo.

In addition to these oral presentations, there are several interesting poster presentations for EV-related studies: 

Lauritzen I at Checler Université Cote d’Azur-CNRS, France, reported enrichment of oligomeric c-terminal fragment of APP called C99 in EVs isolated from C99- or APP-expressing cells or mice. 

Johansson L at Linköping University, Sweden, reported that CRISPR-mediated loss-of-function mutation in VSP35 in SH-SY5Y human neuronal cell line resulted in intracellular accumulation of Ab oligomer, reduction in exosome release, and upregulation of autophagocytic response. Exosomes collected from Ab oligomer-treated SH-SY5Y cells with VPS35 mutation showed neurotoxicity following incubation with organotypic hippocampal slices, suggesting the potential role of the neurotoxic effect. 

Yao P from Kapogiannis laboratory at NIH reported NEVs from AD cases have reduced levels of mitochondrial respiratory protein components compared to controls in two different cohorts, suggesting their reduced energy metabolism. 

Another paper by Eren E from Kapogiannis laboratory reports ante-mortem NEV Ab42 levels are positively associated with ante-mortem cognition and negatively associated with AD Braak stage at autopsy. 

Peng K at Nathan S. Klein Institute, NY, reported enhanced EV production in the brain of APOE2 knock-in mice compared to APOE3 knock-in mice by aging, suggesting enhanced clearance of endosomal molecules by EV secretion in APOE2 knock-in mice. 

Vacchi E at Università della Svizzera Italiana, Switzerland, presented the immune profiling of EVs isolated from plasma and CSF samples of PD, atypical PD with multiple system atrophy, or tauopathies and control cases. Multiple immune makers were differentially expressed in these groups in both plasma- and CSF-derived EVs and can be utilized for discriminating patients from controls with high accuracy and sensitivity. 

Balaguer JS at University of Barcelona presented a study comparing EVs isolated from cortical neurons of SD rat, C57BL/6 mouse, and RTP801 knockout mouse embryonic brains. RTP801/REDD1 is a pro-apoptotic molecule, and its upregulation has been reported in neurodegenerative disorders. EVs from WT neurons enhanced consolidation of glutamatergic synapses in recipient neurons, while deletion of RTP801 reduced EV secretion and its consolidation effect.

Finally, Clayton K from Ikezu lab at Boston University presented enhanced tau propagation from the entorhinal cortex to dentate gyrus in aged APPNL-G-F mice, which developed compact amyloid plaque formation, compared to non-tg mice. This effect was significantly diminished by microglial depletion by PLX5622. A novel microglia-specific expression of mEmerald-CD9 reporter showed enhanced EV secretion from Clec7A+ neurodegenerative microglia compared to homeostatic microglia. Microglia-specific EVs contained p-tau, as determined by super-resolution confocal microscopy and immunoelectron microscopy. 

## DECLARATIONS

### Copyright

© The Author(s) 2021.
